# Beta burst characteristics predict instantaneous hand movements in Parkinson’s disease

**DOI:** 10.1016/j.nbd.2025.107157

**Published:** 2025-10-25

**Authors:** Zuhair Hawa, Koorosh Mirpour, Jeong Woo Choi, Nader Pouratian

**Affiliations:** Department of Neurological Surgery, University of Texas Southwestern Medical Center, Dallas, TX 75390, USA.

**Keywords:** Parkinson’s disease, Bradykinesia, Kinematics, Electrocorticography, Hand movement, Beta burst

## Abstract

Parkinson’s disease (PD) is a neurodegenerative disorder characterized by bradykinesia, rigidity, and tremor. Amplification of beta-band oscillations in both cortical and subcortical areas have often been implicated in the pathoetiology of PD symptomatology. Although previous work has linked prolonged beta bursts to motor impairment, few studies have examined the moment-by-moment causal role of beta bursting in driving movement kinematics. We hypothesize that beta burst characteristics predict instantaneous hand movement dynamics and may, therefore, play a fundamental role in the pathophysiology of disease and guiding. We analyzed the characteristics of beta burst dynamics within and between cortical and subcortical regions and their causal relationship with hand kinematics. We recorded neural and hand-movement data from the motor (M1), premotor (PM), and internal Globus Pallidus (GPi) of 20 PD patients during deep brain stimulation (DBS) implantation surgery. We used Generalized Linear Models and Linear Mixed-Effects Models to identify how beta burst characteristics, including power, duration, and bursting rate, predict instantaneous hand motion.

High beta burst duration, power, and bursting rate in M1, PM, and GPi correlated with movement kinematics, with significant interactions noted between high beta burst dynamics in GPi and low beta power in M1 (*p* < 0.05). Collectively, these parameters predicted hand motion 35.6 % of the time. The findings underscore that both low- and high-frequency beta oscillations across interconnected cortical–subcortical networks contribute to bradykinesia and dynamically influence movement kinematics. By delineating the temporal and spectral parameters that predict hand motion, our work advances the understanding of bradykinesia’s neural underpinnings and suggests new opportunities for targeted closed-loop therapeutic interventions.

## 1. Introduction

Parkinson’s disease (PD) is a progressive neurodegenerative disorder primarily characterized by rigidity, tremor, and bradykinesia, with the latter presenting as difficulty initiating movement and a gradual reduction in movement speed and amplitude during repetitive tasks (Rodriguez-Oroz et al., 2009; Ling et al., 2012). At the physiological level, bradykinesia has been linked to heightened beta power in both cortical and subcortical structures such as subthalamic nucleus (STN) and globus pallidus internus (GPi), as well as increased synchrony of beta-band activity within and across these regions (Steiner et al., 2017; Cagnan et al., 2015).

In particular, transient increases in the amplitude of beta oscillations—often referred to as beta bursts—have emerged as a key feature of pathophysiological underpinning of PD motor symptomatology (Tinkhauser et al., 2018; Lofredi et al., 2019; Mirpour and Pouratian, 2024). Prolonged beta burst duration in the basal ganglia (BG) is a disease-specific characteristic in PD and that dopaminergic medication can shorten these bursts (Lofredi et al., 2019; Kehnemouyi et al., 2020; Kondylis et al., 2016; O’Keeffe et al., 2020a; Duchet et al., 2021). This suggests that shorter beta bursts may represent a healthier non-diseased physiological pattern. Beta burst power has also been implicated, showing that an increase in this measure is associated with an increase in symptom severity (Radcliffe et al., 2023). Patients with PD have also been noted to have a reduction in inter-burst intervals in the beta band (Heideman et al., 2020). Most investigations to date, however, have assessed correlations between brain activity and behavior over long time-blocks without addressing the dynamic moment-by-moment fluctuations in both beta oscillations and movement kinematics, to better understand the dynamic interactions between brain and behavior. More recent work has begun to examine the real-time evolution of neural signals, highlighting the need to capture transient fluctuations in beta power and phase that may underlie movement initiation and execution (Lofredi et al., 2019; Radcliffe et al., 2023). Such temporally resolved analyses could more directly implicate physiological phenomenon in the pathophysiology of disease and symptomatology in PD.

Beyond limitations imposed by studying relationships over long blocks of time, many studies have treated the beta band as a single functional entity despite growing electrophysiological evidence supports a functional distinction between low-beta (~13–20 Hz) and high-beta (~20–35 Hz) sub-bands (van Wijk et al., 2016; Tsiokos et al., 2017; Oswal et al., 2021; Boon et al., 2019; Binns et al., 2024). In PD, amplified low-beta activity in the STN and GPi has been consistently reported and shown to be attenuated by dopaminergic medication or deep brain stimulation (DBS) (Malekmohammadi et al., 2018). Notably, therapeutic modulation of low-beta activity correlates with motor symptom improvement (Duchet et al., 2021). Since effective motor control depends on the suppression of low-beta oscillations within basal ganglia-cortical (BGC) motor network (van Wijk et al., 2017; Choi et al., 2020), their pathological amplification in PD likely reflects impaired network dynamics underlying motor deficits (Radcliffe et al., 2023). In contrast, high-beta activity appears more relevant to BGC coupling in PD, as treatments reduce high-beta coherence between the BG and cortex (Malekmohammadi et al., 2018; Oswal et al., 2013). Computational models further suggest that cortical high-beta activity propagates via the hyperdirect pathway to generate altered low-beta oscillations in the BG (Oswal et al., 2021). These findings suggest that low- and high-beta oscillations may contribute to PD pathophysiology at different spatial scales—local (subcortical) and global (cortico-subcortical), respectively. However, how these transient alterations within BGC motor network are associated with actual kinematics in PD remains a knowledge gap. Demonstrating causality among the network nodes by electrophysiological signals in human is inherently challenging, but constructing a predictive model can approximate causal inferences by ensuring that the predictor (neural activity) precedes the outcome (behavior). To that end, characterizing beta burst dynamics to predict kinematics provides a framework for investigating how fluctuations in brain activity drive bradykinetic features of PD.

Against this background, our study aims to evaluate whether specific characteristics of beta bursts, including burst power, burst duration, burst rate, and inter-burst interval within BGC network, are associated with movement kinematics in PD. We focus on distinguishing low- and high-beta frequencies, motivated by their distinct roles abovementioned. We hypothesize that high-beta bursts within the nodes of the motor BGC networks are associated with low-beta bursts in BG during movements, which can help predict the changes in hand movement parameters. We recruited PD patients undergoing DBS implantation targeting GPi to take advantage of the unique opportunity to record the signals from the BGC networks nodes such as motor cortex (M1), premotor cortex (PM), and GPi, simultaneously, while they were performing repeated hand opening/closing movements. By determining the optimal timing of neural measurements relative to instantaneous hand movement, we aim to develop a predictive model of bradykinesia that connects neural signaling within BGC networks to the resulting motor output. Through this model, we expect to demonstrate a putative causal influence of beta burst dynamics on bradykinesia, thereby advancing our understanding of the mechanisms of altered beta oscillatory activities in PD.

## 2. Methods

### 2.1. Surgical procedure

This study was approved by the Institutional Review Boards of the University of California, Los Angeles. All patients provided written informed consent in accordance with the Declaration of Helsinki. A total of 20 patients with PD (6 female, 14 male, average age of 64.4 ± 6.7 years) who were scheduled for DBS implantation surgery participated in this study (Table 1). The average off medication UPDRS-III score was 38 ± 15. Patients were assessed in a practical off state, having discontinued all PD medications for at least 12 h before the surgical procedure to minimize confounding effects of dopaminergic treatment. All DBS implantations were carried by a single surgeon and conducted due to clinical necessity. Leads were implanted bilaterally in the GPi per previous studies in our group (Mirpour and Pouratian, 2024; Malekmohammadi et al., 2018; O’Keeffe et al., 2020b). The GPi was targeted based on multidisciplinary clinical review. We specifically aimed to study GPi because it is implicated in both the direct and indirect motor pathways and serves as the primary output nucleus of the motor circuit directly modulating the thalamocortical pathways that drive movement. The subthalamic nucleus (STN) is of great interest across studies, but does not reflect the final output of the basal ganglia and does not provide insights into the contributions from the direct pathway. An eight-contact, subdural electrocorticography (ECoG) strip with 4 mm platinum-iridium electrodes (AdTech Medical) was inserted temporarily through the burr hole created for the insertion of the DBS lead. Locations of the electrodes were determined pre-operatively using previously described techniques and the surgical steps following previously published protocol (Mirpour and Pouratian, 2024; Malekmohammadi et al., 2018; O’Keeffe et al., 2020b).

### 2.2. Electrophysiological and behavioral data recordings

Electrophysiological recordings were made using BCI2000 V3 software through two designated recording g.USBamp amplifiers (g.Tech, Austria). For all electrodes, a single scalp reference was used per previous studies from our lab (Mirpour and Pouratian, 2024; Malekmohammadi et al., 2018; O’Keeffe et al., 2020b). These recordings were made at a sampling rate of 2400 Hz and bandpass filtered at 0.1 Hz to 1000 Hz. The electrode immediately anterior to the central sulcus was deemed to be recording from motor cortex. The electrode anterior to this one was designated as recording from premotor cortex. Anatomical registration of the ECoG strip on the subject-specific brain was determined using methods described in previous reports, projecting intraoperative fluoroscopic images of the skull and array projected onto the cortical surface reconstructed from the preoperative MRI and preoperative/postoperative CT scans (Mirpour and Pouratian, 2024; Malekmohammadi et al., 2018; O’Keeffe et al., 2020b).

DBS electrode leads were implanted using methods previously described by our lab (Mirpour and Pouratian, 2024; Malekmohammadi et al., 2018; O’Keeffe et al., 2020b). A Leksell stereotactic headframe (Elekta Instruments) was mounted to the skull followed by a head CT scan. The DBS electrode (Model 3387, 1.27 mm lead body diameter, contact length 1.5 mm, inter-contact distance 1.5 mm, Medtronic Inc.) were implanted into the GPi using image-guided targeting and preplanned using individualized MRI scans.

**Fig. 1.**
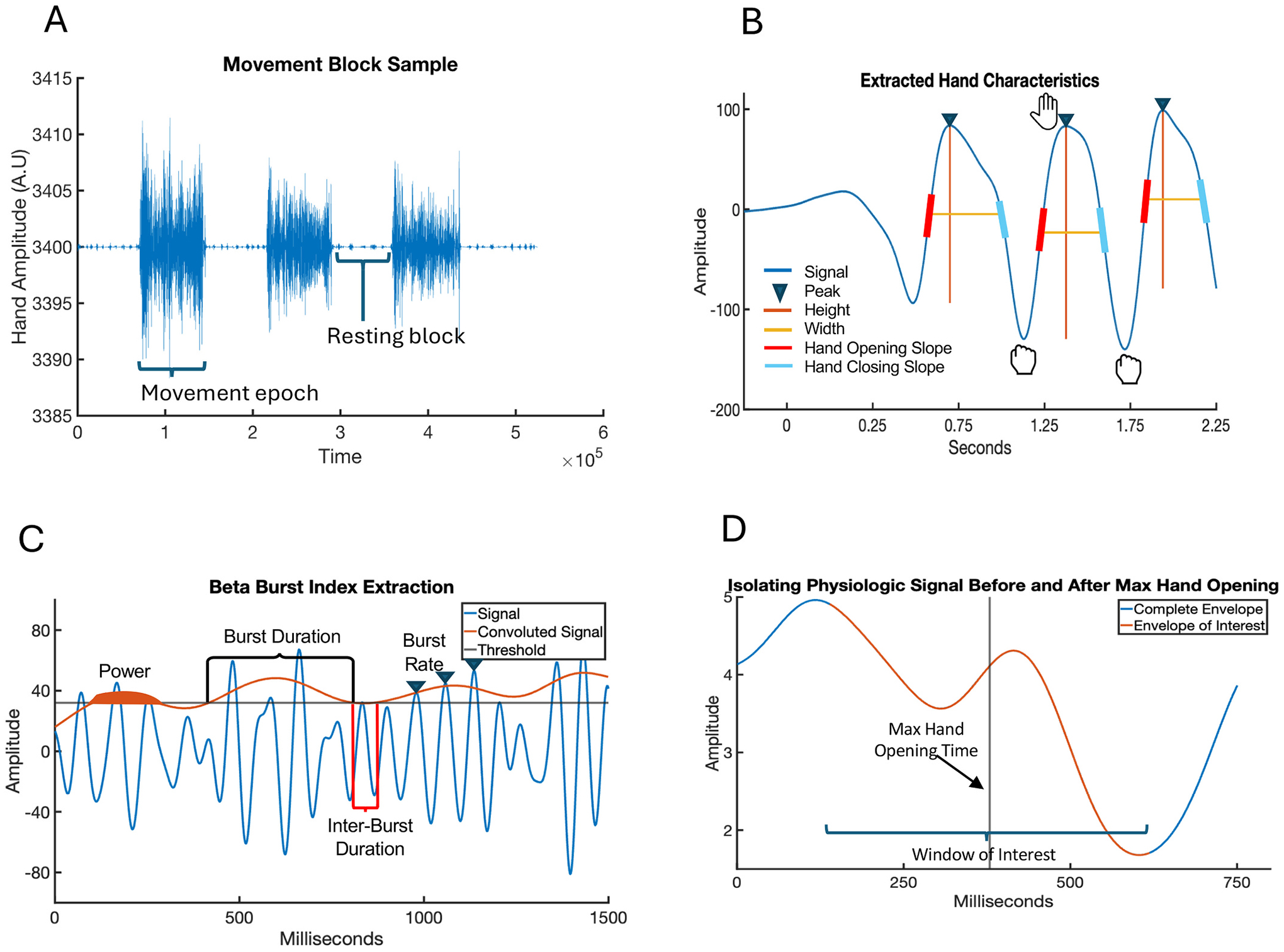
Experimental Design (A) Example of an epoch-design recording session showing hand-movement amplitude (blue trace) over time. The movement epoch is the area with increased amplitude as marked and the resting period is the region with a lower overall amplitude in comparison. (B) Illustration of how the hand movement characteristics were extracted. The triangle at the peak denotes a complete cycle, whose vertical orange line represents the maximum hand-opening magnitude and whose horizontal yellow line indicates the cycle width (the time required to fully open and close the hand). The red ascending slope reflects the opening speed, while the black descending slope reflects the closing speed. (C) Neural beta-band characteristics extraction. The blue trace is the narrow-band signal of interest, and the orange line is its analytical envelope. The black horizontal line marks the 75th percentile amplitude threshold for detecting beta bursts. A beta burst is defined as any interval where the envelope exceeds this threshold. Burst duration is the time the signal remains above threshold; burst rate is the number of envelope peaks above threshold within a burst; and inter-burst duration is the time elapsed between consecutive bursts. (D) Isolation of neural data with respect to maximum hand opening. The blue line shows the analytical envelope of the neural signal. The vertical black line marks the time of maximum hand opening, and the orange trace highlights the portion of the signal analyzed over a window starting at 100 ms centered at this event and increasing in 100 ms steps up to 500 ms.

Prior to recording, patients were woken from anesthesia and were allowed to recover from propofol for at least 30 min as per previous reports (Mirpour and Pouratian, 2024; Malekmohammadi et al., 2018; O’Keeffe et al., 2020b). Once ready, hand movements were measured using a glove containing individual finger piezoelectric sensors. Patients performed repeated cycles (minimum 3, maximum 10) of a 30-s hand-opening-and-closing task, followed by 30 s of rest (Fig. 1A). Each movement epoch consisted of sequential hand openings and closings (Fig. 1B), where the index finger sensor served as a proxy for the entire hand. Only the movement periods (i.e., the 30-s hand-opening-and-closing epochs) were included in subsequent analyses. Electrophysiological recordings from both the ECoG strip and DBS electrodes along with the hand movement recordings were time-synchronized.

### 2.3. Hand movement analysis

All hand movement analysis were completed offline using custom MATLAB scripts (Mathworks Inc., NA, USA). The glove signals were filtered with a 1–7 Hz bandpass filter. For each cycle (opening and closing of the hand), we extracted four metrics (illustrated in Fig. 1B), including 1) amplitude: maximum hand opening (peak of the hand signal); 2) cycle width: time from the start of the opening to the completion of the closing (time of one opening-closing cycle); 3) increasing slope: speed of hand opening (slope from start to peak); and 4) decreasing slope: speed of hand closing (slope from peak to the end of closing).

### 2.4. Neural signal analysis

All electrophysiological recordings were analyzed offline using custom MATLAB scripts. Recordings were bandpass-filtered using a Butterworth bandpass filter into two beta sub-bands: low beta (12–20 Hz) and high beta (21–35 Hz) (Malekmohammadi et al., 2018). A Hilbert transform was applied to each sub-band signal to compute its analytic amplitude envelope. Beta bursts were identified when the amplitude envelope exceeded the 75th percentile of its distribution (Fig. 1C) for each recording site across all movement epochs per patient. In addition, the threshold was calculated separately for PM, M1, and GPi. Bursts shorter than 50 ms were excluded as this would be less than half of the fastest beta oscillation, consistent with previous reports (Mirpour and Pouratian, 2024). We calculated four specific burst characteristics: 1) the burst duration: the length of time each burst exceeded the threshold; 2) burst power: amplitude integrated over the duration of each burst; 3) burst rate: the number of bursts per unit time; and 4) inter-burst interval: the time between the end of one burst and the start of the next.

### 2.5. Modeling hand movements

To identify the best time window for quantifying movement dynamics, we evaluated 5 time windows (100, 200, 300, 400, and 500 ms), each centered at the time of maximal hand opening (Fig. 1D). If a burst began or ended outside the window, the portion outside the window was excluded. A generalized linear model (GLM) was used to evaluate the relationship between burst characteristics for each time window and hand movement metrics within that window (i.e., the kinematic feature of interest). One GLM was developed individually for the M1, PM, and GPi locations at each frequency band for each time window. Therefore, for each time window, a total of 6 GLMs (3 BGC nodes × 2 sub-beta bands) were evaluated. The inputs of the each GLM were the four beta burst characteristics (burst duration, burst power, burst rate, and inter-burst interval) that were contained within the defined the time window size. Each GLM had the following overall equation, where the individual letters represent the coefficients: hand movement = a*burst duration + b*burst power + c*burst rate + d*inter-burst interval. Any burst characteristics outside the window were excluded. Hand movements were consolidated into a single variable using a principal component analysis (PCA) to avoid redundancy within the system. Since multiple hand movement parameters are strongly correlated with notable correlation coefficients including those between cycle time and max opening (*r* = 0.16, *P* < 0.001), cycle time and speed of opening (*r* = −0.12, P < 0.001), cycle time and speed of closing (*r* = 0.13, P < 0.001), max opening and speed of opening (*r* = 0.85, *P* < 0.001, max opening and speed of closing (*r* = −0.84, P < 0.001), speed of opening and speed of closing (*r* = −0.97, P < 0.001), which results can be found in Table 2, PCA analysis can generate a single variable. Our PCA analysis revealed that the first PC explained 82.5 % of the variance while the second PC explained 12.5 %. For interpretation purposes, the first PC correlated the strongest with cycle time (R^2^ = 0.5, P < 0.001) while all other hand movements had an R^2^ < 0.05. Since the first PC increased primarily with cycle time, the GLM results can also be interpreted in the context of cycle time. Akaike Information Criterion (AIC) values were averaged over 6 GLMs as a measure of the average predictive error within each time window. A single AIC value allowed for easy comparison between the various time windows. The optimal time window was chosen based on the lowest AIC value.

**Fig. 2.**
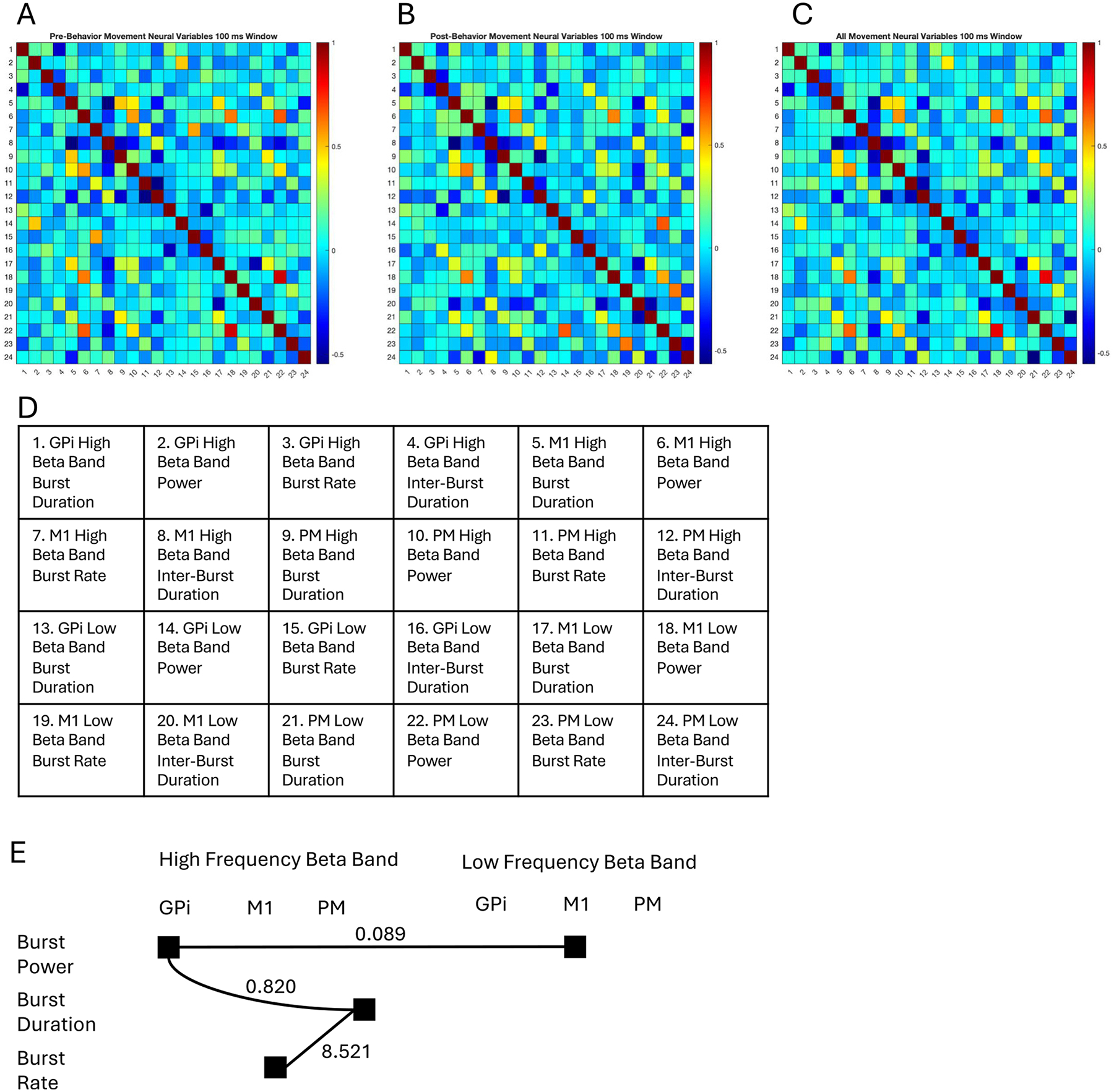
C Experimental Results. These matrices cross-correlate burst characteristics in the GPi, PM, and M1 cortices in (A) pre-behavior, (B) post-behavior and (C) all movement neural variables in a 100 ms window. The numbers on each axis represent different BGC nodes, beta band frequency, and burst characteristic combinations. The colors represent the relative strength of correlation with dark blue representing an R^2^ of −0.5 and maroon representing an R^2^ of 1. (D) Labels of each number. This table includes all the labels for the previous diagrams. (E) Schematic of predictive equation. This schematic shows the different parts of the brain, beta-band characteristics, and beta-band frequencies and how they interact with one another. The square represents the significant location and the line between them indicates their interaction. The coefficient of each interaction is written next to the line. GPi: Globus Palladus Internal, M1: motor cortex, PM: premotor cortex.

We then employed a linear mixed-effects model (LME) to incorporate inter-subject and inter-epoch variability. To prevent model overfitting, a cross-correlation matrix was generated to verify if any burst characteristics with strong inter-correlation (R^2^ > 0.9) were not included in generating the LME (Fig. 2). All beta burst characteristics at both frequency bands in all the BGC nodes were included in a singular LME equation along with any interactions between burst characteristics or BGC nodes. The first model used all 24 main effects (2 sub-beta bands × 4 burst characteristics × 3 BGC nodes) and their 276 pair-wise interactions as the input variables. After running the model, we selected the burst characteristics and BGC nodes that individually demonstrated statistical significance at *P* < 0.05. We then used these significant variables to generate a new model and repeated the selection process. This specialized selection process was completed until the final LME model only had significant variables. These final characteristics were utilized for the predictive model.

### 2.6. Statistical analysis

For the final predictive model created from the LME as described above, we randomly divided the dataset into training (90 %) and testing (10 %) subsets, where 90 % of the hand movement cycles were training data and the remaining 10 % of cycles became the testing data. We generated a model using the training data, fit it to the testing data, and correlated the predicted hand movement to the real hand movement. This correlation was then compared to the baseline correlation established by shuffling the testing data 1000 times. A Student’s *t*-test with an alpha level of 0.05 was used to determine whether the model’s predictions were statistically significant and if so, this was deemed a correct prediction. We ran this for 500 randomly separated training and testing data sets. The model’s final performance was reported as the percentage of correct predictions of the 500 random sets. To assess generalizability of the model, we conducted a leave-one-subject-out cross validation (LOSO-CV) analysis. Instead of randomly separating the training and testing data sets, we used one subject as the testing data and all the others as the training data. Each iteration had a different subject used as the testing subject. All data were preprocessed using custom MATLAB scripts.

## 3. Results

To determine the optimal time window size to define instantaneous hand movement, five time-window sizes evaluated for building the model, and the 100-ms window had the lowest averaged AIC value (8.91 × 10 (Cagnan et al., 2015)) when used in the GLM. All averaged AIC values for the different time window sizes are reported in Table 3. When we developed the GLMs with the 100-ms time window size, we found that only the low-beta burst power in GPi and M1 were each individually statistically significant (*P* < 0.05) and contributed heavily to the low AIC value. Detailed results are presented in Table 4 with all of the corresponding GLM coefficients with asterisks marked on individual coefficients that are *P* < 0.05.

Before constructing our LME model, a cross-correlation analysis confirmed that no neurophysiological parameters were strongly correlated with each other (R^2^ > 0.9, Fig. 2A–C). After including all burst characteristics and both frequency bands and all BGC nodes in the original model, we reduced the final model to the following interactions: GPi high beta band burst power × motor cortex low beta band burst power, GPi high beta band burst power × premotor cortex high beta band burst duration, and motor cortex high beta band burst rate × premotor cortex high beta band burst duration. All of these are statistically significant at *P* < 0.05. These interactions are summarized in Fig. 2E. We additionally generated LME models utilizing other hand parameters such as the maximum hand opening, speed of hand opening, and speed of hand closing to determine if any clinical correlation exists among these parameters. We found that no significant interactions are present when using any other hand parameters.

We also shifted the 100-ms window to determine its optimal location. We created three types of 100-ms windows: 1) all 100-ms occurred before the point of maximal hand opening of the hand signal, 2) 100-ms window centered around the point of maximal hand opening, and 3) all 100-ms after the point of maximal hand opening. Centering the 100-ms window maximal hand opening yielded the lowest overall AIC value (*2087.5*) compared with the other windows. The complete model coefficients and *P* values are listed in Table 5.

### 3.1. Model performance

After the creation of the final LME, we tested its predictive ability. We generated the training model and fit it to the testing data. We found that of the 500 separate trials, 35.6 % of them resulted in a statistically significant prediction (*P* < 0.05). We determined that our model using the LOSO-CV methodology was generalizable to untrained subjects in 3.33 % of untrained cases.

## 4. Discussion

Instantaneous hand kinematics (with hand opening and closing) are dependent on moment-to-moment changes in neurophysiological oscillations in GPi, M1, and PM to predict instantaneous hand movement in patients with PD. Our results demonstrate that the model can predict movement in 35.6 % of our trials, a promising indicator that neural signaling in both cortical and subcortical regions influence hand movement. However, our model could only generalize and predict 3.33 % of untrained subject’s hand movement. Importantly, we determined that a 100-ms window, centered around the maximal hand opening, optimally represents “instantaneous” hand movement.

The predictive model developed in this study supports the potential framework that synchronized high-beta bursts between the cortex and basal ganglia serve as a critical feature of PD pathophysiology which contribute to anti-kinetic states through low-beta activity amplification. We first identified significant interactions among high-beta burst features within nodes of BGC network that were predictive of hand kinematics. Specifically, the relationships between both GPi high-beta burst power and hand kinematics, and M1 high-beta burst rate and hand kinematics, were significantly modulated by the duration of high-beta bursts in the PM. Longer high-beta bursts in the PM were associated with a stronger correlation between M1 high-beta burst rate and hand kinematics, whereas shorter PM high-beta bursts were linked to a stronger correlation between GPi high-beta burst power and hand kinematics. These findings suggest that PM may function as a regulatory node, potentially modulating pallidocortical communication in the high-beta band. This interpretation is consistent with the established roles of PM in movement planning and gating, acting upstream of both M1 and subcortical motor structures (Playford et al., 1993; Blandini et al., 2000; Dum and Strick, 2005). Importantly, the primary behavioral variable outcome predicted by these network interactions—the first PC of hand kinematics—predominantly captured total hand opening and closing time which are particularly sensitive to anti-kinetic states in PD (Slutzky and Flint, 2017). These results underscore the functional relevance of transient high-beta dynamics within the BGC network and support the broader notion that aberrant pallidocortical high-beta synchrony contributes to clinically meaningful motor impairment in PD (Oswal et al., 2016; Tinkhauser et al., 2017; Little and Brown, 2012).

Second, we identified a significant interaction between high-beta burst power in GPi and low-beta burst power in M1 in predicting hand kinematics. Specifically, increased high-beta burst power in GPi was associated with a stronger correlation between M1 low-beta burst power and bradykinetic hand movements. Given that the GPi serves as the primary output structure of the basal ganglia (BG), regulating movement through inhibitory projections to M1—presumably within the beta frequency range (Broussolle et al., 1999; Wichmann and DeLong, 2003)—we posit that elevated GPi high-beta bursts contribute to pathological motor states in Parkinson’s disease (PD) by amplifying M1 low-beta bursts. Notably, the GPi exhibited interactions not only with M1 in the low-beta band but also with PM in the high-beta band. While the exact mechanisms remain to be elucidated, these observations raise the possibility that the GPi may functionally coordinate distinct beta sub-bands across cortical regions, potentially acting as a bridge between high-beta and low-beta dynamics. This aligns with recent computational modeling work, which suggests that the pathological low-beta oscillations in the GPi may arise via STN-GP feedback loop (likely involving the GPe) following the cortical transmission of high-beta oscillations (Oswal et al., 2021). Taken together, our findings support a model in which a cascade of transient high-beta oscillatory activity across the BGC network contributes to an increase in M1 low-beta power, ultimately prolonging hand open-to-close time, exacerbating motor symptoms in PD (Tinkhauser et al., 2017; Little and Brown, 2012).

Our model allows us to predict hand motion in people from whom the data was collected, but is not as accurate in predict movements in people whose data who were not used in the training, indicating that the algorithm is dependent on individual-specific data to successfully predict movement kinematics. This suggests that future clinical translation would require integration of patient-specific data to optimize predictive ability. This could theoretically be obtained intraoperatively during DBS implantation to generate an algorithm that is applied in the postoperative clinic setting.

Despite these promising results, several limitations must be considered. First, the dataset was derived from a relatively small cohort of patients, as recordings occurred during surgical procedures with strict clinical constraints. Consequently, the range of potential hand movements was limited. The available data may thus underrepresent the complexity of patients’ everyday motor behavior. While we purposefully evaluated GPi because of its position as the final common output of the basal ganglia, this target-specific sampling does not enable us to comment on whether the results are GPi-specific or a more generalizable phenomenon of neurophysiology across bagal ganglia nuclei. Further investigations of similar phenomena with STN reocrdings could potentially capture a more holistic circuit model that more precisely sheds light on cause and effect parameters. Moreover, we did not have access to a healthy control group for comparison, restricting our ability to generalize or parse which features are specifically pathological versus normal. Finally, our predictive model employed a linear framework; given the inherent complexity of neural systems, a nonlinear approach may unveil additional predictive power and more accurately capture the dynamics of PD-related movement patterns. Future work will require more naturalistic movements to confirm generalizability and utility. Furthermore, even though we used a data-driven approach to find the optimal time window to define “instantaneous” hand movement, other temporal anchors or dynamic time-warping approaches could optimize model generation and reveal new neural-behavioral associations. Such investigations could also assess the impact of medication or DBS therapy on the observed beta dynamics, potentially leading to refined, patient-specific models. Incorporating a larger patient population and a wider array of behavioral tasks would not only strengthen model robustness but also improve understanding of how these neural signatures evolve dynamically as the patient moves, helping demystify motion development and adjustment in the brain. Moreover, advanced machine learning algorithms or deep learning may capture nonlinear interactions between burst duration, burst power, frequency bands, and neural synchrony.

Our findings underscore the critical role of beta burst dynamics across low and high frequency bands in driving movement kinematics in PD. By disentangling the specific contributions of burst duration and power, as well as elucidating critical cortical–subcortical interactions, we offer new insights into the pathophysiological basis of bradykinesia. The predictive framework presented here marks a significant step towards understanding the exact order of physiological aberrations that lead to bradykinesia. Our study serves as a critical preliminary step for generating a predictive model to accurately capture the complexities of bradykinesia; however additional studies will help improve the accuracy of the model with more naturalistic behaviors and additional neural parameters.

## Figures and Tables

**Table 1:** Patient Demographics. This table is a breakdown of all the patients’ demographics that were included in this study. F stands for female, M stands for male, R stands for right, and L stands for left.

Subject Number	Age (years)	Sex	Handedness	UPDRS-III Score
1	78	F	R	16
2	65	M	R	16
3	63	M	R	51
4	76	F	R	29
5	72	M	R	54
6	67	F	R	42
7	58	M	R	72
9	60	M	R	23
10	70	M	L	42
11	61	F	R	37
13	57	F	R	32
14	64	F	R	34
15	61	M	R	16
16	63	M	R	55
17	72	M	R	31
18	59	M	R	29
19	60	M	R	35
20	52	M	R	57
21	69	M	R	52
22	61	M	R	37

**Table 2:** Cross-correlation of All Hand Parameters. This table has the cross-correlation outcomes for all the hand parameters with each other. All parameters are statistically significant with *P* < 0.001.

	Cycle Time	Max Opening	Speed of Opening	Speed of Closing
Cycle Time		0.17	−0.10	0.11
Max Opening	0.17		0.86	−0.85
Speed of Opening	−0.10	0.86		−0.97
Speed of Closing	0.11	−0.85	−0.97	

**Table 3:** Optimizing Instantaneous Hand Movement. This table shows the outcomes for optimizing the window of interest to define instantaneous hand movement.

Window Size (ms)	Average AIC	Number of Significant Coefficients
100	8.91 × 10^4^	2
200	9.15 × 10^4^	1
300	9.20 × 10^4^	1
400	9.33 × 10^4^	2
500	9.36 × 10^4^	2

**Table 4:** Neurophysiological Parameter GLM for 100 millisecond Time Window. This table shows the coefficients for each neural parameter in each beta band and location. Any coefficients that are individually significant with P < 0.05 are marked with an asterisk. The *P*-value located at the bottom of each column represents the significance of the overall GLM for each BGC node and frequency band combination. GPi: Globus Palladus Internal. AIC: Akaike Information Criterion.

	GPi High Beta Band	Motor High Beta Band	Premotor High Beta Band	GPi Low Beta Band	Motor Low Beta Band	Premotor Low Beta Band
Burst Duration	−1.26	0.69	5.64	11.06	−1.21	3.99
Power	2.18	4.75	9.15	15.22*	23.88*	4.45
Burst Rate	−2.99	1.69	5.24	−7.35	−10.1	−2.58
Inter-Burst Duration	5.18	17.48	35.3	36.01	34.36	−3.15
R^2^	7.00 × 10^−3^	0.014	0.031	0.039	0.047	1.50 × 10^−2^
P-Value	0.59	0.26	0.01	0.002	4.04 × 10^−4^	2.60 × 10^−1^
AIC	9.40 × 10^4^	9.40 × 10 (Cagnan et al., 2015)	8.88 × 10 (Cagnan et al., 2015)	8.90 × 10 (Cagnan et al., 2015)	8.48 × 10 (Cagnan et al., 2015)	8.38 × 10 (Cagnan et al., 2015)

**Table 5:** Optimization of Linear Mixed Effect Model. This table shows the different interactions that occurs for the different window parameters. The interactions shown all are significant with P < 0.05. GPi: Globus Palladus Internal.

Window Location	AIC	Significant Interactions
Pre-behavior	2103.6	None
Post-behavior	2092.6	None
Through behavior	2087.5	0.820 * GPi High Beta Band Power * Premotor Cortex High Beta Band Burst Duration 8.521 * Motor Cortex High Beta Band Burst Rate * Premotor Cortex High Beta Band Burst Duration 0.089 * GPi High Beta Band Power * Motor Cortex Low Beta Band Power

## Data Availability

Data will be made available on request.
